# Dysbiosis of Gut Microbiota and Metabolite Phenylacetylglutamine in Coronary Artery Disease Patients With Stent Stenosis

**DOI:** 10.3389/fcvm.2022.832092

**Published:** 2022-03-25

**Authors:** Chen Fang, Kun Zuo, Yuan Fu, Jing Li, Hongjiang Wang, Li Xu, Xinchun Yang

**Affiliations:** Heart Center and Beijing Key Laboratory of Hypertension, Beijing Chaoyang Hospital, Capital Medical University, Beijing, China

**Keywords:** in-stent stenosis, gut microbiota, phenylacetylglutamine, 16S rRNA, coronary artery disease

## Abstract

**Introduction:**

Dysbiotic gut microbiota (GM) plays a regulatory role during the pathogenesis of several cardiovascular diseases, including atherosclerosis. GM-derived metabolite phenylacetylglutamine (PAGln) enhances platelet responsiveness and thrombosis potential, thereby inducing major adverse cardiovascular events. However, the role of GM and microbial metabolite PAGln in the pathogenesis of in-stent stenosis remains unknown.

**Methods:**

16S rRNA sequencing was performed on fecal samples in 103 coronary artery disease (CAD) patients, including 35 individuals with in-stent patency (control), 32 individuals with in-stent hyperplasia (ISH), and 36 subjects with in-stent stenosis (ISS), and the levels of plasma PAGln were evaluated by enzyme-linked immunosorbent assay.

**Results:**

The results revealed significantly enhanced microbial diversity and disrupted composition, such as enrichment of *Roseburia, Blautia*, and *Ruminococcus*, were observed in CAD patients with in-stent stenosis. The imbalance of microbial function related to PAGln synthesis and elevated plasma GM-derived metabolite PAGln levels was detected in CAD patients with in-stent stenosis. The GM-dependent diagnostic model could identify CAD patients with in-stent stenosis.

**Conclusion:**

The current study revealed the disordered signature, altered functions, and potential diagnostic ability of GM in CAD patients with in-stent hyperplasia and stenosis. Enhanced microbiota-derived PAGln synthesis-related functions and elevated plasma PAGln levels were associated with in-stent stenosis and hyperplasia in CAD patients. Thus, an intervention targeting gut microbes may be a promising strategy to prevent stent stenosis in patients with CAD.

## Introduction

Coronary artery disease (CAD) is the most common public health problem that can culminate in heart attack and is a major cause of morbidity and mortality worldwide (1). Besides pharmacological treatments such as antiplatelet drugs, statin, etc., invasive coronary angiography and revascularization are one of the treatment regimens aimed to restore blood flow, while percutaneous coronary intervention (PCI) is the primary revascularization treatment of CAD (2). However, recent statistics from the National Cardiovascular Data Registry show that approximately 10% of current PCIs were performed for in-stent restenoses (ISR) (3). Treatment of ISR with PCI can vary in complexity but can often be a challenging procedure and outcomes are worse than those compared to the PCI of *de novo* lesions (3). The potential pathogenesis may refer to elastic recoil of the vessel wall after standard balloon angioplasty, the inflammatory response triggered by the vascular injury, and the activation of the proliferation and migration of smooth muscle cells (4). Moreover, the creation of an extracellular matrix ultimately leads to the formation of neointimal tissue, which, in case of excessive hyperplasia, can cause restenosis (4). Therefore, aggressive neointimal proliferation and late neoatherosclerosis might be the recognized aetiopathogenesis (5–7).

Trillions of microbiota colonize in the human intestine, forming a complex physiological ecosystem that regulates host homeostasis (8). Accumulating evidence has confirmed the importance of disordered GM in the development of CAD and atherosclerosis (8, 9). Furthermore, gut microbiota functions as an endocrine organ, producing bioactive metabolites that influence the pathophysiological processes of cardiovascular disease (8). Therefore, blood metabolite serves as a liquid conveyor for molecules derived from GM. Recently, a gut microbially generated metabolite, phenylacetylglutamine (PAGln), has been indicated as a metabolite linked with enhanced platelet (PLT) responsiveness and thrombogenic potential (10, 11). Recent studies suggested that plasma PAGln is associated with coronary atherosclerotic burden and an increased future risk of CAD (12, 13).

In light of the above, we assume that disrupted GM composition, altered GM function, and the GM-derived PAGln might be associated with the pathogenesis of in-stent hyperplasia and stenosis in CAD patients. Hence, in this study, 16s sequencing was performed to address the potential changes of GM composition and functions and the subsequent effects on clinical progression from in-stent patency, hyperplasia to stenosis. In addition, this study evaluated the plasma PAGln levels for its potential association with in-stent stenosis in CAD patients.

## Materials and Methods

### Study Subjects and Baseline Clinical Characteristics

A total of 103 CAD patients hospitalized for rechecking coronary angiography in the Beijing Chaoyang Hospital between September 2020 and July 2021 and treated with successful PCI for at least 1 year were enrolled in this study. The participants included 35 with in-stent patency, 32 with in-stent intimal hyperplasia, and 36 with in-stent stenosis. The exclusion criteria were as follows: use of antibiotics or probiotics in the past 1 month, acute or chronic infection, severe liver and kidney dysfunction, autoimmune disease, hematological system disease, malignant tumors, or mental illness. All subjects received regular secondary preventive treatment for CAD. The study was approved by the ethics committee of Beijing Chaoyang Hospital. The research protocol conformed to the principles of the Declaration of Helsinki. All subjects signed the informed consent.

The clinical characteristics of all patients, including age, gender, body mass index (BMI), history of PCI, smoking and drinking, medical history, and medication, were recorded. Also, left ventricular ejection fraction (LVEF), blood routine, serum lipid levels, liver, and kidney function, and hemoglobin A1c (HbA1c) were examined.

### Fecal and Blood Sample Collection

Fresh fecal samples and peripheral venous blood were collected from each subject in the morning after admission. The stool samples were frozen and stored at −80°C immediately. Plasma was isolated from blood samples by centrifugation at 3,000 rpm, 4°C for 10 min and stored at −80°C until further analysis.

### 16S rRNA Sequencing

Microbial DNA was extracted using TIANGEN kit. After amplification, purification and quantification, 16S rRNA community profiles were measured using Illumina MiSeq sequencing of the V4 region (200–450 bp) according to the manufacturer's instructions. Sequence denoising or operational taxonomic unit (OTU) clustering was performed according to QIIME2 DADA2 analysis process or Vsearch software analysis process. The GreenGene Database was used to annotate taxonomic information.

### Plasma PAGln Quantification

The protocol determined plasma PAGln concentrations using enzyme-linked immunosorbent assay (ELISA) kits (ml520101, MLBio, China).

### Statistical Analysis

Continuous variables with or without normal distribution were represented as the mean ± standard deviation (SD) or median (quartile), and categorical variables were represented as the number (percentage). Continuous variables in the normal distribution or non-normal distribution were analyzed by *t*-test or Mann–Whitney test, respectively. Categorical variables were analyzed using the χ^2^ test. Statistical analyses were performed using SPSS version 25.0 (IBM Corp., Armonk, NY, USA).

The Chao richness, Simpson index, Shannon index, and Pielou evenness were calculated at the genera levels using QIIME2. Principle co-ordinate analysis (PCoA) was carried out using related packages in R software (version 2.15.3). Statistical variation in gut microbial composition was evaluated using the analysis of similarity (ANOSIM). The linear discriminant analysis (LDA) effect size (LEfSe) analyses were performed on the Galaxy online platform (http://huttenhower.sph.harvard.edu/galaxy). The cutoff value was the absolute LDA score (log 10) >2.0. The differential abundance of genus and KEGG orthology was calculated by Wilcoxon rank-sum test, and *P*-values were corrected by Benjamin and Hochberg multiple tests. Pearson's correlation coefficients were calculated in R software (Version 2.15.3). The envfit analysis based on the Bray-Curtis distance was used to calculate the effect size and significance of clinical parameters on the total GM variation. The receiver operating characteristic (ROC) was analyzed using MedCalc statistical software. The partial least squares structural equation modeling (PLS-SEM) analysis was conducted by SmartPLS 3 software. The least absolute shrinkage and selection operator (LASSO) analysis and Random forest algorithm were performed using R software (version 2.15.3). *P* < 0.05 (two-sided) was regarded as statistically significant.

## Results

### Clinical Characteristics of Patients

In this study, 103 CAD patients after coronary stent implantation for at least 1 year and regular medication were enrolled in this study. The cohort included 35 patients with in-stent patency (control) (80.00% male; age 65.60 ± 8.30 years), 32 with in-stent intimal hyperplasia (ISH) (81.25% male; age 64.06 ± 10.67 years), and 36 with in-stent stenosis (ISS) (75.00% male; age 63.22 ± 8.92 years). The clinical characteristics and laboratory values of CAD patients in three groups are represented in Table 1. No significant difference was detected in age, time of stent implantation, gender, hypertension (HTN), diabetes mellitus (DM), smoking history, drinking history, BMI, TC, TG, AST, ALT, serum creatinine, HbA1c, LVEF, WBC and HGB between control and ISH or ISS groups. Compared to the control group, patients in the ISS group had higher PLT levels (*P* = 0.016), while no significant difference was detected in the ISH group (*P* = 0.321).

**Table 1:** Baseline clinical characteristics of participants in study.

	**In-stent patency (Control)**	**In-stent intimal hyperplasia (ISH)**	**In-stent stenosis (ISS)**	***P*-value (C *vs*. ISH)**	***P*-value (C *vs*. ISS)**
Number	35	32	36	—	—
Age, years	65.60 ± 8.30	64.06 ± 10.67	63.22 ± 8.92	0.511	0.249
Time of stent implantation, years	5.00 (4.00, 8.00)	5.50 (2.00, 9.00)	6.50 (3.25, 8.75)	0.965	0.367
Male (%)	28 (80.00)	26 (81.25)	27 (75.00)	1.000	0.778
HTN (%)	24 (68.57)	17 (53.13)	28 (77.78)	0.219	0.430
DM (%)	20 (57.14)	16 (50.00)	15 (41.67)	0.628	0.238
Smoking (%)	13 (37.14)	10 (31.25)	21 (58.33)	0.797	0.098
Drinking (%)	9 (25.71)	11 (34.38)	15 (41.67)	0.594	0.211
BMI, kg/m^2^	25.45 ± 2.99	27.00 ± 4.10	25.61 ± 3.29	0.096	0.843
TC, mmol/L	3.41 ± 0.66	3.20 ± 0.71	3.41 ± 0.72	0.203	0.989
HDL-C, mmol/L	0.98 ± 0.25	0.91 ± 0.28	0.98 ± 0.21	0.300	0.959
LDL-C, mmol/L	1.98 ± 0.62	1.81 ± 0.59	1.93 ± 0.67	0.235	0.752
TG, mmol/L	1.09 (0.91, 1.57)	1.22 (0.89, 1.80)	1.26 (1.02, 2.14)	0.547	0.241
AST, U/L	20.00 (16.00, 27.00)	20.18 ± 5.10	18.00 (16.00, 21.00)	0.312	0.149
ALT, U/L	18.00 (15.00, 32.00)	22.38 ± 10.69	18.00 (14.00, 25.75)	0.722	0.417
SCr, μmol/L	68.74 ± 13.31	70.40 (62.63, 76.60)	69.28 ± 12.84	0.312	0.863
HbA1c (%)	6.55 (5.70, 7.43)	6.70 ± 1.02	6.40 (5.90, 7.73)	0.620	0.397
LVEF (%)	66.00 (60.00, 70.00)	63.00 (60.00, 70.00)	64.03 ± 9.27	0.655	0.820
WBC, (× 10^9^/L)	6.08 ± 1.44	5.94 (5.15,7.49)	6.66 ± 1.46	0.427	0.097
HGB, (× 10^9^/L)	132.17 ± 15.12	129.50 ± 17.62	135.06 ± 16.97	0.507	0.453
PLT, (× 10^9^/L)	189.00 (176.00, 212.00)	207.00 (174.50, 230.00)	226.00 ± 46.81	0.321	0.016

*Data are shown as mean ± SD, median (quartile) or number (percentage). ALT, alanine aminotransfease; AST, aspartate aminotransferase; BMI, body mass index; DM, diabetes mellitus; HTN, hypertension; HGB, hemoglobin; HbA1c, hemoglobin A1c; HDL-C, high-density lipoprotein cholesterol; LVEF, left ventricular ejection fraction; LDL-C, low density lipoprotein cholesterol; PLT, platelet; SCr, serum creatinine; TC, total cholesterol; TG, triglyceride; WBC, white blood cell*.

### Alterations in Gut Microbial Diversity and Enterotype

**Figure 1:**
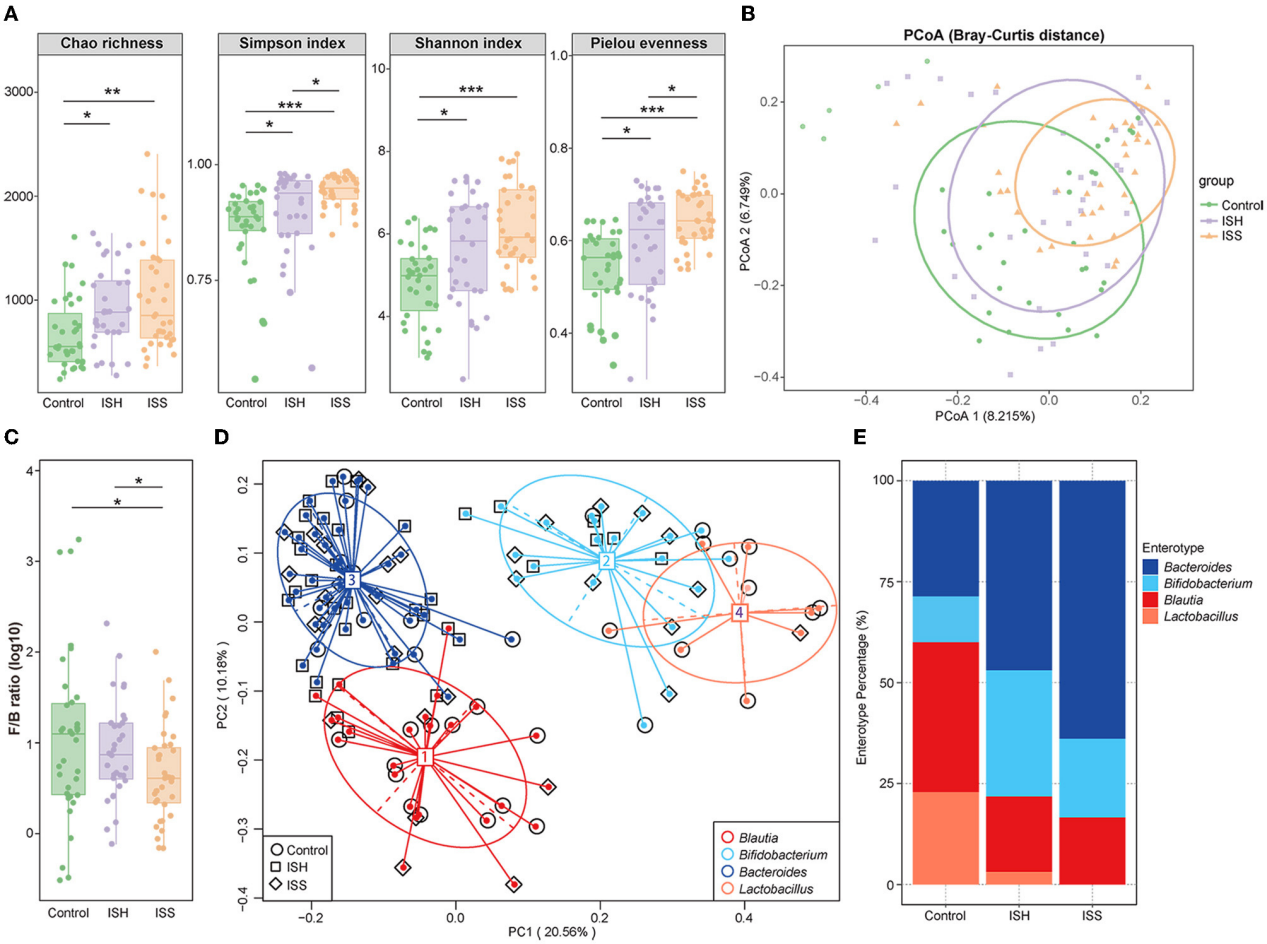
Altered gut microbial diversity and enterotypes in CAD patients with ISH and ISS. **(A)** Within-individual (alpha) diversity includes Chao richness, Simpson index, Shannon index, and Pielou evenness based on the genera profile in the control, ISH, and ISS groups. **(B)** PCoA of beta-diversity analysis based on Bray–Curtis distance show separation of the genera in the control, ISH, and ISS groups. **(C)** The *Firmicutes*/*Bacteroidetes* (F/B) ratio in the control, ISS, and ISH patients with CAD. (*P* = 0.798, Control *vs*. ISH; *P* = 0.036, ISH *vs*. ISS; *P* = 0.035, C *vs*. ISS). **(D)** A total of 103 samples from the control, ISH, and ISS groups are stratified into four enterotypes by PCoA of Jensen–Shannon divergence at the genera level. The major contributor in the four enterotypes is *Blautia, Bifidobacterium, Bacteroides*, and *Lactobacillus*. **(E)** The percentage of control, ISH, and ISS samples is distributed in the four enterotypes. *, *P* < 0.05; **, *P* < 0.01; ***, *P* < 0.001; *n* = 35 for the control group, *n* = 32 for the ISH group, *n* = 36 for the ISS group; Wilcoxon rank-sum test.

The composition of GM was examined using 16S rRNA sequencing in CAD patients who had undergone PCI with in-stent patency, hyperplasia, and stenosis. Compared to CAD patients with in-stent patency, significant gut microbial alterations were detected in patients with ISH and ISS. Different from the control patients, the within-individual (alpha) diversity, including Chao richness, Simpson index, Shannon index, and Pielou evenness, exerted a remarkably increasing trend in the ISH and ISS patients (Control *vs*. ISH group: *P* = 0.012 for Chao richness, *P* = 0.015 for Simpson index, *P* = 0.013 for Shannon index, *P* = 0.020 for Pielou evenness; Control *vs*. ISS group: *P* = 0.003 for Chao richness, *P* < 0.001 for Simpson index, *P* < 0.001 for Shannon index, *P* < 0.001 for Pieou evenness) (Figure 1A). Higher Chao, Simpson, Shannon, and Pielou indices were recorded in ISS patients than ISH patients (Figure 1A). However, no statistically significant differences were detected in Chao and Shannon indices. Furthermore, PCoA analysis showed that the controls and ISS patients cluster into different groups but fail to dramatically distinguish ISS patients from ISH patients (Figure 1B). The results of ANOSIM analysis indicated the significant difference in the GM composition among the three groups (Supplementary Figure S1A; ANOSIM, r = 0.083, *P* = 0.001). To determine whether clinical factors exerted an additional effect on the gut microbial composition, the amount of variance (r^2^) explained by each clinical characteristics based on envfit analysis and the result showed that DM exerted significant correlation (envfit analysis, r^2^ = 0.176, *P* = 0.04) (Supplementary Figure S1B).

To further identify the altered microbial structure at the different states of the stent after PCI in CAD patients, we analyzed the microbial enterotype features using the Partitioning Around Medoid (PAM) clustering method. Then, PCoA analysis divided 103 samples into four clusters, dominated by *Blautia, Bifidobacterium, Bacteroides*, and *Lactobacillus*, respectively. Remarkable alterations were observed in enterotype distribution (Figure 1D). Compared to the controls, CAD patients with ISH and ISS possessed a higher percentage of *Bacteroides* and *Bifidobacterium* and a lower percentage of *Blautia* and *Lactobacillus* (Figure 1E). Thus, stent status in CAD patients after PCI is associated with altered gut microbial communities with a tendency toward the enterotype dominated by *Bacteroides* and *Bifidobacterium*, while those away from the enterotype consist of *Blautia* and *Lactobacillus*. These results indicated a potential influence of gut microbes in the progression of in-stent stenosis in CAD patients who underwent PCI.

### Differences in Taxonomic Profiles Between Patients With ISS and Controls

**Figure 2:**
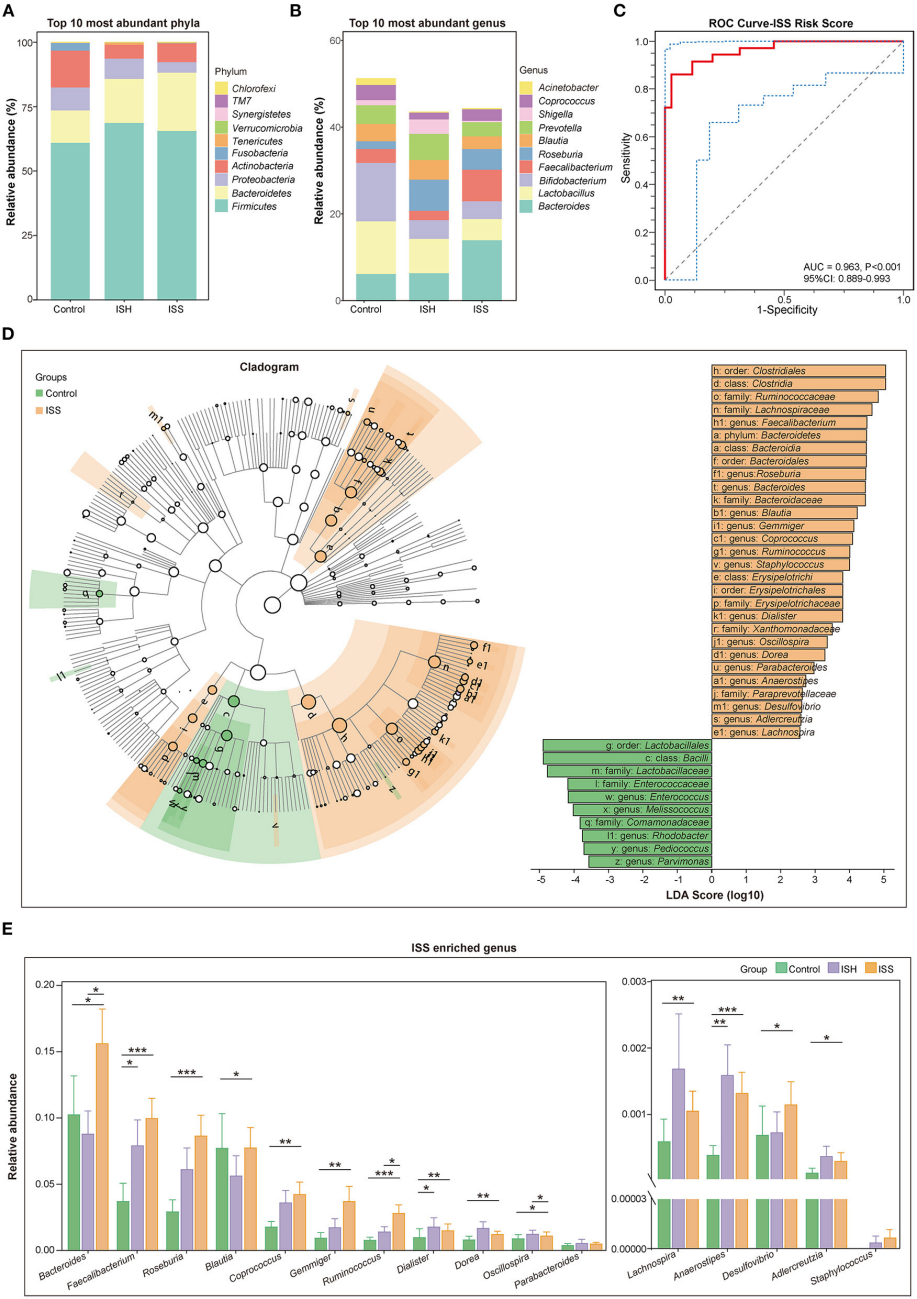
Distinct difference in gut microbiota in the CAD patients with in-stent patency, hyperplasia, and stenosis. **(A)** Bar plot of relative abundance of the top 10 most abundant phyla in the control, ISH, and ISS groups. **(B)** Bar plot of relative abundance of the top 10 most abundant genera in the control, ISH, and ISS groups. **(C)** The ROC curve for the ISS risk score to verify the predicted capability of the model (AUC = 0.963, *P* < 0.001, 95% CI: 0.889–0.993). **(D)** Right panel; cladogram reveals different taxonomic compositions between the control (green) and ISS (orange) groups. Left panel; LDA coupled with effect size shows differentially enriched microbiota at all taxonomic levels in the control and ISS groups at the threshold of absolute LDA score (log 10) >2.0. **(E)** The bar chart shows the relative abundance of the genera enriched in the ISS group. *, *P* < 0.05; **, *P* < 0.01; ***, *P* < 0.001; Wilcoxon rank-sum test. *n* = 35 for the control group, *n* = 32 for the ISH group, *n* = 36 for the ISS group.

The taxonomic profiles of the GM among patients in control, ISH, and ISS groups were evaluated. As shown in Figure 2A, the relative abundance of the top 10 most abundant phyla included *Firmicutes, Bacteroidetes, Proteobacteria, Actinobacteria, Fusobacteria, Tenericutes, Verrucomicrobia, Synergistetes, TM7*, and *Chlorofexi*. Several studies suggested that the disordered ratio of *Firmicutes*/*Bacteroidetes* (F/B ratio) is a characteristic of various diseases (14). Compared to control patients, CAD patients with ISH and ISS had a progressively decreased F/B ratio (Figure 1C). The difference between controls and ISS patients was statistically significant (*P* = 0.035). The top 10 most enriched genus of each group, such as *Bacteroides, Lactobacillus, Bifidobacterium, Faecalibacterium, Roseburia, Blautia, Prevotella, Shigella, Coprococcus*, and *Acinetobacter* are represented in Figure 2B. The high abundance of *Bacteroides, Faecalibacterium*, and *Roseburia*, and low abundance of *Lactobacillus, Bifidobacterium, Blautia, Prevotella, Shigella, Corprococcus*, and *Acinetobacter* was detected in CAD patients with in-stent stenosis. LEfSe analysis was performed to further determine differentially enriched GM between samples and explore the biomarkers for gut microbes in CAD patients with ISS. Interestingly, the GM between the two groups was altered profoundly (Figure 2D). The enrichment of genera *Faecalibacterim, Roseburia, Bacteroides, Blautia, Gemmiger, Coprococcus, Ruminococcus, Staphylococcus, Dialister, Oscillospira, Dorea, Parabacteroides, Anaerostipes, Desulfovibrio, Adlercreutzia*, and *Lachnospira* is associated with in-stent stenosis in CAD patients (Figure 2D). Moreover, the genera *Enterococcus, Melissococcus, Rhodobacter, Pediococcus*, and *Parvimonas* were significantly abundant in CAD patients with in-stent patency (Figure 2D). Notably, as shown in Figure 2E, these microbial genera exerted similar shifted trends in CAD patients with in-stent hyperplasia. Thus, the dysbiosis of intestinal flora is involved in the progression of in-stent stenosis after PCI in CAD patients, including the initial process-stent intimal hyperplasia.

### In-stent Stenosis Predictive Model Based on GM and Clinical Features

In order to identify CAD patients with a high risk of stent stenosis after PCI, we established a predictive model based on gut microbial and clinical signatures. Based on the LASSO analysis, genera *Faecalibacterium, Roseburia, Coprococcus, Gemmiger, Ruminococcus, Enterococcus, Anaerostipes, Adlercreutzia*, and *Bacteroides*, smoking, TG, and PLT were selected as the predictors for the occurrence of in-stent stenosis in CAD patients treated with PCI. The in-stent stenosis risk score was calculated as follows: ISS Risk Score = [−12.140 × (Intercept)] + [1.410 × (*Faecalibacterium*)] + [13.340 × (*Roseburia*)] + [31.350 × (*Coprococcus*)] + [94.630 × (*Gemmiger*)] + [45.460 × (*Ruminococcus*)] + [−3582.000 × (*Enterococcus*t)] + [85.740 × (*Anaerostipes*)] + [2894.000 × (*Adlercreutzia*)] + [4.474 × (*Bacteroides*)] + [0.601 × (smoking)] + [0.671 × (TG)] + [0.032 × (PLT)]. Smoking was counted as one point. CAD patients in the ISS group had dramatically high-risk scores (*P* < 0.001) (Supplementary Figure S2C). ROC analysis was performed to validate the predictive risk model. The sensitivity, specificity, and AUC were 86.11, 97.14, and 96.30% (95% CI: 0.889–0.993, *P* < 0.001), respectively (Figure 2C). Furthermore, based on random forest algorithm, the top seven important variables were selected to construct a risk prediction model, of which five variables were identical to the predictors selected by the LASSO analysis (Supplementary Figures S1C–F). In the testing data set (30%), the AUC of this model is 0.867 (95% CI: 0.680–1.000). Therefore, the ISS risk score constructed using LASSO analysis was chosen for further analysis owing to its higher predictive potential.

### Functional Alterations Related to PAGln in GM of Patients With In-stent Stenosis

**Figure 3:**
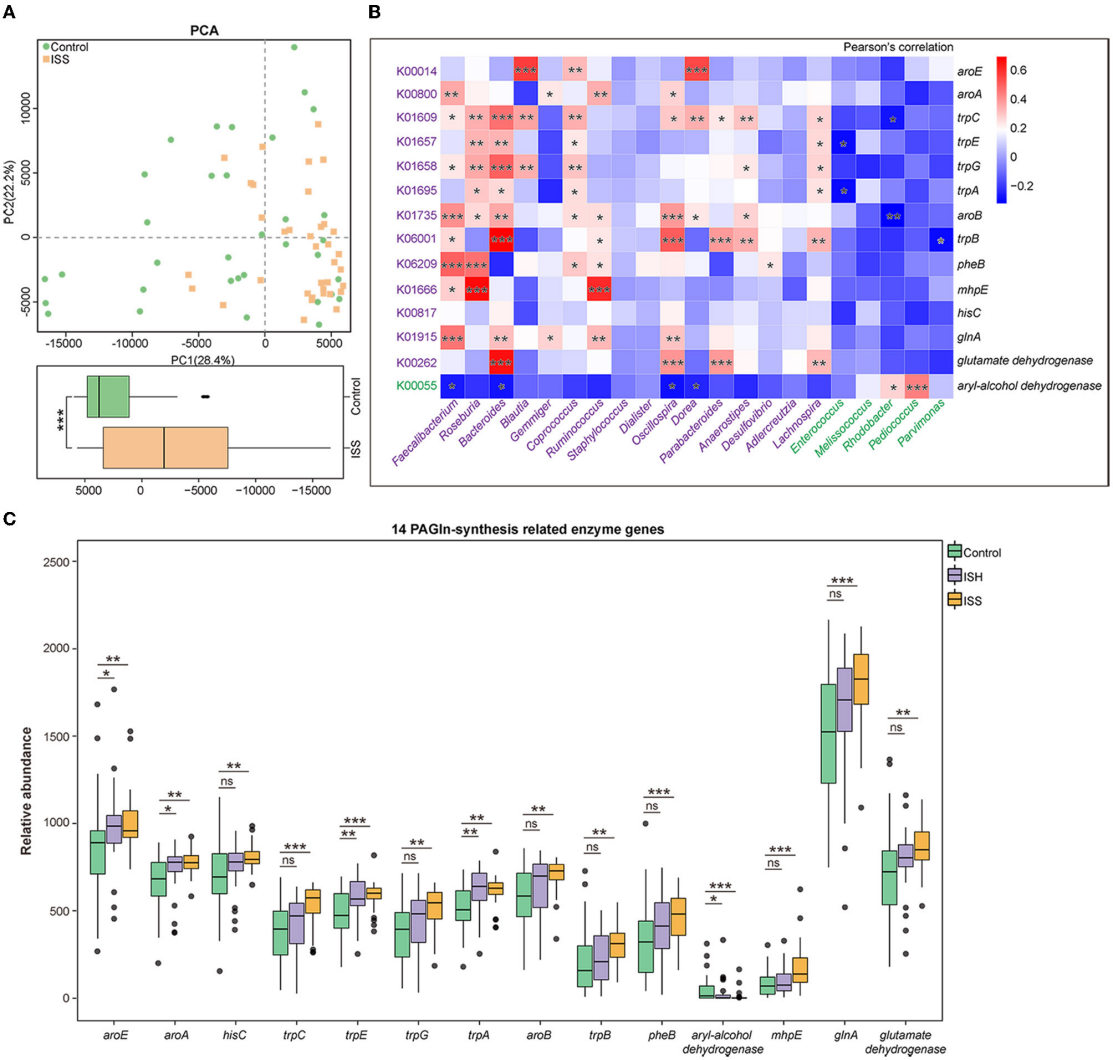
Functional alteration in GM of CAD patients with in-stent patency, hyperplasia, and stenosis. **(A)** PCA based on the relative abundance of KOs shows different functions of gut microbiota in the Control and ISS groups. The box plot shows a significant difference at the first principal component (PC1) values between the control and ISS groups. Wilcoxon rank-sum test. **(B)** Heatmap shows the correlation between 14 PAGln-synthesis related KOs/enzyme genes and GM abundant in the control and ISS groups, respectively, via Pearson's correlation analysis. **(C)** The box plot reveals the relative abundance of 14 PAGln-synthesis related enzyme genes in the control, ISH, and ISS groups. *, *P* < 0.05; **, *P* < 0.01; ***, *P* < 0.001; ns, not significant; Wilcoxon rank-sum test. *n* = 35 for the control group, *n* = 32 for the ISH group, *n* = 36 for the ISS group.

Based on the Kyoto Encyclopedia of Genes and Genomes (KEGG) database, we evaluated gut bacterial functions in the three groups. The principal component analysis (PCA) analysis on the first principal component (PC1) revealed significant differences in the microbial function between the control and ISS groups (*P* < 0.001, Wilcoxon rank-sum test) (Figure 3A).

Dietary phenylalanine is metabolized by intestinal flora to generate phenylacetic acid (PAA), which condenses with glycine (Gly) to form PAGln, which is involved in major cardiovascular events, such as myocardial infarction (MI) and stroke (10, 15). Annotation from the KEGG database revealed the KEGG orthology (KO) with striking differences between the control and ISS groups; a total of 14 KOs/enzyme genes participated in the biosynthesis and metabolism of PAA (C07086) and Gly (C00037). Notably, patients in the ISS and ISH groups possessed alterations similar to the controls. Except for higher *aryl-alcohol dehydrogenase* (K00055) in the controls (*P* < 0.001 for control *vs*. ISS group; *P* = 0.036 for control *vs*. ISH group), we found that CAD patients with in-stent stenosis and hyperplasia possessed upregulated enzymatic genes, including *shikimate dehydrogenase* (*aroE*, K00014), *3-phosphoshikimate 1-carboxyvinyltransferase* (*aroA*, K00800), *histidinol-phosphate aminotransferase* (*hisC*, K00817), *indole-3-glycerol phosphate synthase* (*trpC*, K01609), *anthranilate synthase component I* (*trpE*, K01657), *anthranilate synthase component II* (*trpG*, K01658), *tryptophan synthase alpha chain* (*trpA*, K01695), *3-dehydroquinate synthase* (*aroB*, K01735), *tryptophan synthase beta chain* (*trpB*, K06001), *chorismate mutase* (*pheB*, K06209), *4-hydroxy 2-oxovalerate aldolase* (*mhpE*, K01666), *glutamate dehydrogenase* (K00262), and *glutamine synthetase* (*glnA*, K01915) related to PAGln synthesis in the gut (Figure 3C). The *aryl-alcohol dehydrogenase* (K00055) catalyzes phenylacetaldehyde, an upstream metabolite of PAA, to form phenylethyl alcohol, thereby affecting PAA generation.

Furthermore, the correlation between 14 gut PAGln-related synthesis factors and 21 differentially enriched genus of gut flora in CAD patients with or without in-stent stenosis [|LDA score (log 10)|>2, *P* < 0.05] was evaluated by correlation analysis. As shown in Figure 3B, ISS patients-enriched genera, including *Faecalibacterim, Roseburia, Bacteroides, Blautia, Gemmiger, Coprococcus, Ruminococcus, Staphylococcus, Dialister, Oscillospira, Dorea, Parabacteroides, Anaerostipes, Desulfovibrio, Adlercreutzia*, and *Lachnospira*, were positively correlated with the 13 enzyme genes enriched in CAD patients with ISS, respectively and negatively related to *aryl-alcohol dehydrogenase*, which was enriched in the controls. Conversely, a positive correlation was detected between *aryl-alcohol dehydrogenase* and controls-enriched genera, such as *Melissococcus, Rhodobacter, Pediococcus*, and *Parvimonas*. Thus, alterations in GM composition and related functions are linked to the development of in-stent stenosis in CAD patients after PCI.

### Correlations of Gut Microbiota and Clinical Parameters

**Figure 4:**
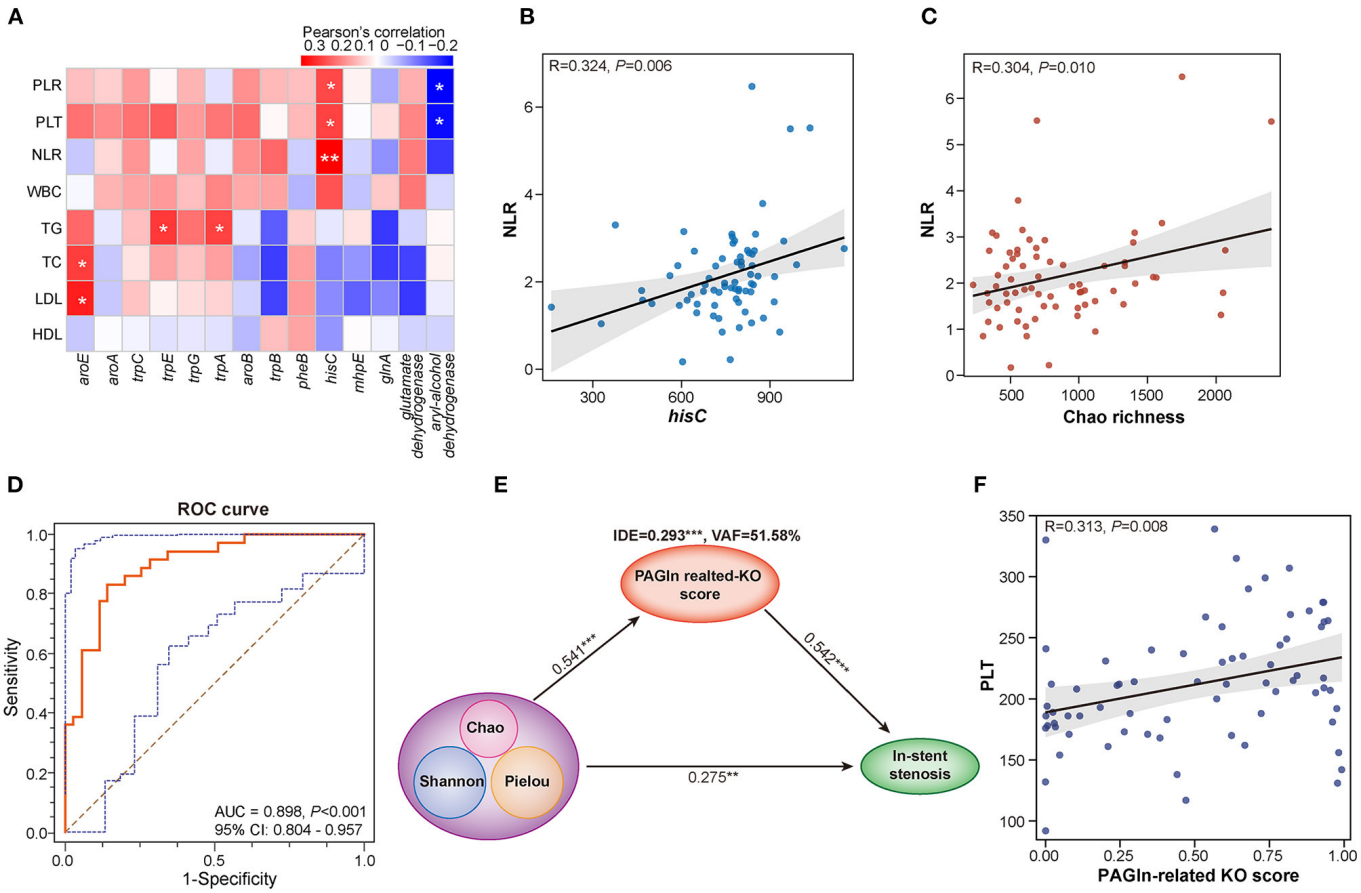
Interactions among gut microbiota, PAGln synthesis-related enzyme genes, clinical parameters, and in-stent stenosis in CAD patients. **(A)** Heatmap shows the Pearson's correlation between PAGln-related synthetic enzyme genes and clinical parameters, including NLR, PLT, PLR, WBC, TG, TC, LDL, and HDL. **(B)** Positive correlation between *hisC* and NLR (R = 0.324, *P* = 0.006). **(C)** Positive correlation between Chao richness and NLR (R = 0.304, *P* = 0.010). **(D)** ROC curve for the KO score based on 14 PAGln-related synthetic enzyme genes/KOs (AUC = 0.898, *P* < 0.001, 95% CI: 0.804–0.957). **(E)** PLS-SEM models show the mediation effect of PAGln-related synthetic function in the total effect of gut microbial diversity on in-stent stenosis in CAD patients. **(F)** Positive correlation between the KO score and PLT (R = 0.313, *P* = 0.008). *, *P* < 0.05; **, *P* < 0.01; ***, *P* < 0.001; *n* = 35 for the control group, *n* = 36 for the ISS group.

NLR and PLR are calculated as the absolute count of neutrophils and PLT divided by the absolute count of lymphocytes, respectively. Both are reliable inflammatory biomarkers and are involved in several diseases, such as atherosclerosis, acute coronary syndromes, CAD, hypertension, and obesity (16–18). The calculation of Pearson's correlation coefficients revealed that Chao richness was positively correlated with NLR (R = 0.304, *P* = 0.010) and PLR (R = 0.162, *P* = 0.177) in CAD patients with or without in-stent stenosis (Figure 4C and Supplementary Figure S2F). The abundant 14 PAGln-synthesis related microbial enzyme genes in the ISS group were positively associated with PLR, NLR, PLT, and WBC (Figure 4A). Especially, *hisC*, an enzyme referred to several steps of PAA synthesis, is remarkably correlated with NLR (R = 0.324, *P* = 0.006) and PLR (R = 0.237, *P* = 0.046) (Figure 4B and Supplementary Figure S2E). Moreover, *aroE* was positively related to LDL-C (R = 0.284, *P* = 0.017) and TC (R = 0.250, *P* = 0.035), and TG was positively linked to *trpE* (R = 0.255, *P* = 0.032) and *trpA* (R = 0.243, *P* = 0.041). Interestingly, *aryl-alcohol dehydrogenase* was negatively associated with NLR (R = −0.205, *P* = 0.086), PLT (R = −0.262, *P* = 0.027), and PLR (R = −0.276, *P* = 0.020). These results revealed that alterations in gut bacterial composition and microbial PAGln-synthesis related enzyme genes are positively associated with systematic inflammation; both may promote stent stenosis by increasing the inflammatory response.

### Interaction Among Gut Microbes, PAGln-Synthesis Related Microbial Enzyme Genes, and In-stent Stenosis in CAD Patients

To further assess the effect of PAGln synthesis-related microbial functions in the progression of stent stenosis, we calculated the PAGln-related KO score based on a linear combination of 14 PAGln synthesis-related KOs/enzyme genes differentially enriched in the control and ISS groups. The model and score are represented in Supplementary Table S1. CAD patients with ISS had remarkably high KO scores (*P* < 0.001), and the area under the ROC curve (AUC) for the KO score was high (AUC = 0.898, 95% CI: 0.804–0.957, *P* < 0.001) (Figure 4D). A significant positive correlation was established between the KO score and PLT (R = 0.313, *P* = 0.008) in CAD patients with or without ISS (Figure 4F). The partial least squares structural equation modeling (PLS-SEM) analysis suggested that a high KO score mediated a positive indirect effect (IDE) (VAF = 51.58%, β = 0.293, *P* < 0.001) during disordered GM promoted in-stent stenosis in CAD patients (Figure 4E). Moreover, GM dysbiosis also directly influenced the in-stent stenosis development (β = 0.275, *P* = 0.004). Thus, the involvement of GM dysbiosis was partially affected by enhanced PAGln-related synthesis in the in-stent stenosis of CAD.

### Elevated Plasma PAGln Levels in CAD Patients With In-stent Stenosis

**Figure 5:**
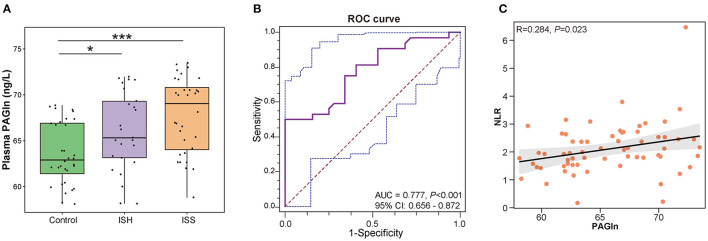
Elevated plasma PAGln levels in CAD patients with in-stent patency, hyperplasia, and stenosis. **(A)** Higher plasma PAGln levels in the ISH and ISS groups than the controls. Control *vs*. ISH, *P* = 0.028; ISH *vs*. ISS, *P* = 0.128; *, *P* < 0.05; ***, *P* < 0.001; Wilcoxon rank-sum test; *n* = 32 for the control group, *n* = 25 for the ISH group, *n* = 32 for the ISS group. **(B)** ROC curve for plasma PAGln (AUC = 0.777, *P* < 0.001, 95% CI: 0.656–0.872). **(C)** Positive correlation between the plasma PAGln level and NLR (R = 0.284, *P* = 0.023).

We further compared the plasma PAGln levels between CAD patients with in-stent patency, hyperplasia, and stenosis. A total of 14 blood samples were missing from the above 103 participants, and the detailed clinical characteristics are shown in Supplementary Table S2. No statistical differences were observed among the three groups in terms of baseline clinical characteristics. The plasma PAGln levels were significantly higher in the ISH and ISS patients than the controls (*P* = 0.028 for Control *vs*. ISH and *P* < 0.001 for Control *vs*. ISS) (Figure 5A). The AUC for plasma PAGln was 0.777 (95% CI: 0.656–0.872, *P* < 0.001) (Figure 5B). Additionally, plasma PAGln levels were positively correlated with NLR (R = 0.284, *P* = 0.023) (Figure 5C).

## Discussion

A close correlation was established between disordered gut microbes and CVD. The gut microbial dysbiosis contributes to CAD, hypertension, atherosclerosis, and arterial aneurysms. However, the characteristics of gut microbiota in CAD patients with in-stent stenosis are yet unknown. In the present study, we obtained evidence on the correlation between GM alteration and stent stenosis after successful PCI in CAD patients based on 16S rRNA sequencing analysis. The CAD patients with in-stent stenosis exerted remarkably increased gut microbial diversity, and thus, the overgrowth of microbiota in the gut promotes stent stenosis in CAD patients who underwent PCI. The gut microbiota in ISS patients changed from the enterotype dominated by *Blautia* and *Lactobacillus* to that dominated by *Bacteroides* and *Bifidobacterium* and possessed a low ratio of *Firmicutes*/*Bacteroidetes*. A similar altered trend was found in CAD patients with in-stent stenosis and hyperplasia. The in-stent hyperplasia mediates the initial stage of stent stenosis and can be regarded as the prophase of in-stent stenosis. These results demonstrated the close association between gut microbial imbalance and in-stent stenosis in CAD patients. Furthermore, both ISH and ISS individuals could be distinguished from the controls by variations in gut microbial metabolic functions, especially the PAGln-synthesis related function. Patients with ISS and ISH had elevated profiles of PAGln-synthesis related enzyme genes compared to the controls. We also found that plasma PAGln levels were higher in the CAD patients with stent stenosis or hyperplasia than in the controls. This phenomenon hinted that an increase in the specific group of gut microbiota induces microbial metabolic related-function dysbiosis, triggering the accumulation of PAGln in the circulation, which could promote the progression from in-stent hyperplasia to stenosis in CAD patients after PCI. The constructed prediction model based on GM profiling could help identify the CAD patients with a high risk of in-stent stenosis after undergoing PCI in the future.

Previous studies reported that the main biological factors of in-stent stenosis include initial intimal hyperplasia and late neoatherosclerosis (5). PLTs, inflammation, and endothelial dysfunction are also involved in neointimal proliferation and restenosis (19). The imbalance of gut microbiota and related bacterial metabolites, including lipopolysaccharide (LPS), trimethylamine-N-oxide (TMAO) and bile acid, plays direct roles in atherosclerosis, inflammation, and PLT hyperreactivity (20). Accumulating evidence has revealed the association between gut dysbiosis and arterial remodeling. Compared to conventional mice, germ-free mice show attenuated neointimal hyperplasia development and reduced local inflammation in arteries after carotid ligation (21). Concomitantly, CAD patients with in-stent hyperplasia and stenosis showed significant alteration in gut microbiota diversity and composition compared to CAD patients with in-stent patency. Previous studies suggested that the decline in the population of *Bifidobacterium spp*. is correlated with endothelial dysfunction (22). Herein, we found a gradually reduced abundance of genera *Bifidobacterium* in the controls, ISH, and ISS groups. *Ruminococcus* exerts pro-inflammatory effects and promotes the release of interferon (IFN)-γ, interleukin (IL)-17, and IL-22 (23). *Ruminococcus* accelerates the development of type 2 diabetes, inflammatory bowel disease, and atrial fibrillation (AF) (24–26). Dietary α-linolenic acid impairs arterial thrombus formation, tissue factor expression, and PLT activation (27). *Blautia* is negatively correlated with α-linolenic acid in AF patients (28). CAD patients with in-stent stenosis shared the enrichment of genus *Ruminococcus, Blautia, Roseburia, Coprococcus, Dialister*, and *Dorea*, in AF (24). Additionally, GM in ISS patients possessed unique characteristics different from other CVD. *Faecalibacterim* function as a significant anti-inflammatory factor dominant in ISS patients (29). Notably, we identified a similarly altered trend in CAD patients with in-stent stenosis and hyperplasia. The in-stent hyperplasia mediates the initial stage of stent stenosis and can be regarded as the prophase of in-stent stenosis. These results demonstrated a correlation between gut microbial imbalance and in-stent stenosis in CAD patients.

Gut microbiota is a virtual endocrine that acts on distal organs through microbial metabolites and affects their physiology and functions. Recent studies demonstrated that a gut microbiota-derived metabolite, phenylacetylglutamine, enhances PLT activation and thrombosis potential in whole blood, isolated platelets, and animal models of arterial injury by triggering adrenergic receptor (ADR) signaling (10). In CAD patients with ISH and ISS, we found that a total of 13 PAGln-synthesis related enzyme genes were remarkably increased in ISS and ISH patients compared to in-stent patency patients except for *aryl-alcohol dehydrogenase*, which partially interferes with PAGln production. PAGln-synthesis related enzyme genes enriched in ISS patients mediated about 51.58% indirect effect of microbial disruption promoting in-stent stenosis. Moreover, these PAGln-synthesis related enzyme genes abundant in ISS patients were positively correlated to NLR, PLR, PLT, TG, TC, and LDL, respectively, while *aryl-alcohol dehydrogenase* was negatively associated with PLR, NLR, and PLT. Simultaneously, Chao richness was positively correlated to NLR and PLR. The clinical parameters, NLR and PLR, reflected systemic inflammation levels. Next, we confirmed that plasma PAGln levels were significantly elevated in CAD patients with in-stent stenosis and hyperplasia than in patients with stent patency. Thus, disrupted gut microbial structure and function and elevated microbiota-derived PAGln in the circulation caused by increased bacterial PAGln synthase were linked to in-stent stenosis in CAD patients who had undergone PCI. This phenomenon may aggravate the progression of in-stent stenosis by promoting inflammation, platelet response, and endothelial dysfunction. Additional studies, including gut microbiota transplantation and cellular experiments in the future, are required to confirm this hypothesis. The present study revealed that GM dysbiosis and related metabolic PAGln are involved in the development of stent stenosis after PCI and provide treatment with the gut microbiota to prevent stent stenosis.

Although the potential influences of drug use, such as antibiotics, probiotics, statins, aspirin, and clopidogrel, were excluded, diet, exercise, and sleep information were not corrected in this study. In addition, mechanical and operator-related factors also contribute to stent stenosis. The current study was insufficient to analyze and correct these effects. The number of participants was relatively small and the validation cohort might provide additional evidence to strengthen the current results. Thus, large prospective cohorts are needed to clarify this issue.

## Conclusion

The present study revealed the imbalance in the patterns of gut microbes, aberrant microbial functions of PAGln-synthesis, and elevated plasma PAGln levels in CAD patients with in-stent hyperplasia and stenosis. The altered GM was correlated to systemic inflammation. A risk score constructed on gut microbiota and clinical signatures exhibit diagnostic ability in CAD patients with stent stenosis. These findings demonstrated a close association among GM dysbiosis, microbiota-derived PAGln, and stent stenosis in CAD patients. GM regulation is a promising target for preventing stent stenosis in CAD patients. Thus, further studies are needed in the future.

## Data Availability Statement

The data presented in the study are deposited in the Sequence Read Archive (SRA) repository, accession number PRJNA806458.

## Ethics Statement

The studies involving human participants were reviewed and approved by the Ethics Committee of Beijing Chaoyang Hospital. The patients/participants provided their written informed consent to participate in this study.

## Author Contributions

All authors designed and executed the study and wrote the manuscript. All authors read and approved the final manuscript.

## Conflict of Interest

The authors declare that the research was conducted in the absence of any commercial or financial relationships that could be construed as a potential conflict of interest.

## Publisher's Note

All claims expressed in this article are solely those of the authors and do not necessarily represent those of their affiliated organizations, or those of the publisher, the editors and the reviewers. Any product that may be evaluated in this article, or claim that may be made by its manufacturer, is not guaranteed or endorsed by the publisher.

## Supplementary Material

Supplementary Table S1 The model and score of PAGln-related KO Score.

Supplementary Table S2 Baseline clinical characteristics of participants in the study of plasma PAGln.

Supplementary Figure S1 Detailed information of ANOSIM, envfit and random forest analysis.

Supplementary Figure S2 Detailed information of LASSO analysis and correlation between PLR and hisC as well as Chao richness.
