# Supplementary material for: Dysbiosis of Gut Microbiota and Metabolite Phenylacetylglutamine in Coronary Artery Disease Patients With Stent Stenosis
Source: Front Cardiovasc Med. 2022 Mar 25;9:832092. doi: 10.3389/fcvm.2022.832092 (PMC8990098; doi:10.3389/fcvm.2022.832092)
Supplement: Supplementary Table S1 — The model and score of PAGln-related KO Score. [file Table_1.pdf]

Table S1

The model and score of PAGln-related KOs score.

Group	The KO Score	Group	The KO Score
Control	0.240153516	ISS	0.928948044
Control	0.075507648	ISS	0.982238976
Control	0.383591045	ISS	0.591846012
Control	0.605973847	ISS	0.785659940
Control	0.209710971	ISS	0.639450807
Control	0.573633462	ISS	0.820671753
Control	0.019917939	ISS	0.754381709
Control	0.470513575	ISS	0.992156861
Control	0.000511291	ISS	0.407298240
Control	0.246962932	ISS	0.771415627
Control	0.000066800	ISS	0.625969508
Control	0.029513604	ISS	0.667907187
Control	0.283508436	ISS	0.342332236
Control	0.722473196	ISS	0.462073315
Control	0.566600265	ISS	0.592265213
Control	0.005466619	ISS	0.829825013
Control	0.183474688	ISS	0.946331012
Control	0.033423711	ISS	0.961593111
Control	0.047745495	ISS	0.931537642
Control	0.024824178	ISS	0.975709244
Control	0.007023062	ISS	0.679950802
Control	0.114965030	ISS	0.931946384
Control	0.000635918	ISS	0.536583421
Control	0.001121003	ISS	0.739224436
Control	0.104595064	ISS	0.661823141
Control	0.843075119	ISS	0.201404083
Control	0.297148507	ISS	0.623282360
Control	0.001626567	ISS	0.883678494
Control	0.264181903	ISS	0.932958870
Control	0.001596017	ISS	0.924414370
Control	0.509039933	ISS	0.954082462
Control	0.355160554	ISS	0.816992829
Control	0.905065356	ISS	0.079241349
Control	0.440994223	ISS	0.932840199
Control	0.735873623	ISS	0.978365977
		ISS	0.807924307

The KO Score =
[-1.342e+01×(Intercept)]+[-1.714e-03×(K00014)]+[8.377e-03×(K00800)]+[1.507e-02×(K01609)]+[2.032e-02×(K01657)]+[-1.078e-02×(K01658)]+[-1.166e-02×(K01695)

]+[4.715e-03×(K01735)]+[-4.043e-04×(K06001)]+[1.969e-04×(K06209)]+[-6.503e-03×(K00055)]+[3.079e-03×(K00817)]+[4.580e-03×(K01666)]+[1.325e-03×(K01915)]+[-7.072e-03×(K00262)]. K00014, *aroE*; K00800, *aroA*; K01609, *trpC*; K01657, *trpE*; K01658, *trpG*; K01695, *trpA*; K01735; *aroB*; K06001, *trpB*; K06209, *pheB*; K00055, *aryl-alcohol dehydrogenase*; K00817, *hisC*; K01666, *mhpE*; K01915, *glnA*; K00262, *glutamate dehydrogenase*.
