# Supplementary material for: Dysbiosis of Gut Microbiota and Metabolite Phenylacetylglutamine in Coronary Artery Disease Patients With Stent Stenosis
Source: Front Cardiovasc Med. 2022 Mar 25;9:832092. doi: 10.3389/fcvm.2022.832092 (PMC8990098; doi:10.3389/fcvm.2022.832092)
Supplement: Supplementary Table S2 — Baseline clinical characteristics of participants in the study of plasma PAGln. [file Table_2.pdf]

Table S2

Baseline clinical characteristics of participants in the study of plasma PAGln.

	In-stent patency (Control)	In-stent intimal hyperplasia (ISH)	In-stent stenosis (ISS)	*P* value (C *vs* ISH)	*P* value (C *vs* ISS)
Number	32	25	32	—	—
Age, years	65.44 ± 8.46	64.12 ± 10.96	64.22 ± 8.96	0.610	0.578
Time of stent implantation , years	4.50 (3.25, 7.75)	5.00 (1.75, 8.50)	6.00 (3.25, 8.75)	0.936	0.248
Male (%)	27 (84.38)	20 (80.00)	24 (75.00)	0.735	0.536
HTN (%)	22 (68.75)	12 (48.00)	25 (78.13)	0.174	0.572
DM (%)	18 (56.25)	11 (44.00)	14 (43.75)	0.429	0.454
Smoking (%)	13 (40.63)	7 (28.00)	18 (56.25)	0.406	0.317
Drinking (%)	8 (25.00)	8 (32.00)	12 (37.50)	0.570	0.419
BMI, kg/m^2	25.48 ± 3.00	26.94 ± 4.10	25.57 ± 3.37	0.141	0.919
TC, mmol/L	3.41 ± 0.66	3.23 ± 0.76	3.44 ± 0.72	0.369	0.840
HDL-C, mmol/L	0.96 (0.78, 1.08)	0.92 ± 0.30	0.96 (0.78, 1.14)	0.456	0.722
LDL-C, mmol/L	1.97 ± 0.62	1.84 ± 0.62	1.95 ± 0.67	0.460	0.905
TG, mmol/L	1.10 (0.92, 1.54)	1.19 (0.89, 1.77)	1.27 (1.03, 2.29)	0.629	0.181
AST, U/L	20.00 (16.00, 27.75)	19.96 ± 5.36	18.00 (16.00, 21.00)	0.249	0.121
ALT, U/L	20.00 (15.00, 33.50)	22.64 ± 10.72	18.00 (14.00, 25.75)	0.672	0.307
SCr, µmol/L	69.88 ± 13.08	72.35 ± 14.08	69.58 ± 13.12	0.497	0.926
HbA1c (%)	6.50 (5.70, 7.00)	6.65 ± 1.00	6.55 (5.90, 7.75)	0.609	0.313
LVEF (%)	66.50 (60.50, 70.00)	65.00 (61.00, 71.00)	63.06 ± 9.07	0.936	0.548
WBC, (×10^9/L)	6.12 ± 1.44	6.51 ± 1.93	6.64 ± 1.52	0.385	0.164
HGB, (×10^9/L)	132.94 ± 15.51	131.84 ± 15.94	135.13 ± 16.95	0.795	0.592
PLT, (×10^9/L)	191.00 (176.25, 213.50)	206.00 (175.00, 233.50)	224.50 ± 48.96	0.536	0.053

Data are shown as mean ± SD, median (quartile) or number (percentage). ALT, alanine aminotransfease; AST, aspartate aminotransferase; BMI, body mass index; DM, diabetes mellitus; HTN, hypertension; HGB, hemoglobin; HbA1c, hemoglobin A1c; HDL-C, high-density lipoprotein cholesterol; LVEF, left ventricular ejection fraction; LDL-C, low density lipoprotein cholesterol; PLT, platelet; SCr, serum creatinine; TC, total cholesterol; TG, triglyceride; WBC, white blood cell.
