# Supplementary figures and images for: Dysbiosis of Gut Microbiota and Metabolite Phenylacetylglutamine in Coronary Artery Disease Patients With Stent Stenosis
Source: Front Cardiovasc Med. 2022 Mar 25;9:832092. doi: 10.3389/fcvm.2022.832092 (PMC8990098; doi:10.3389/fcvm.2022.832092)

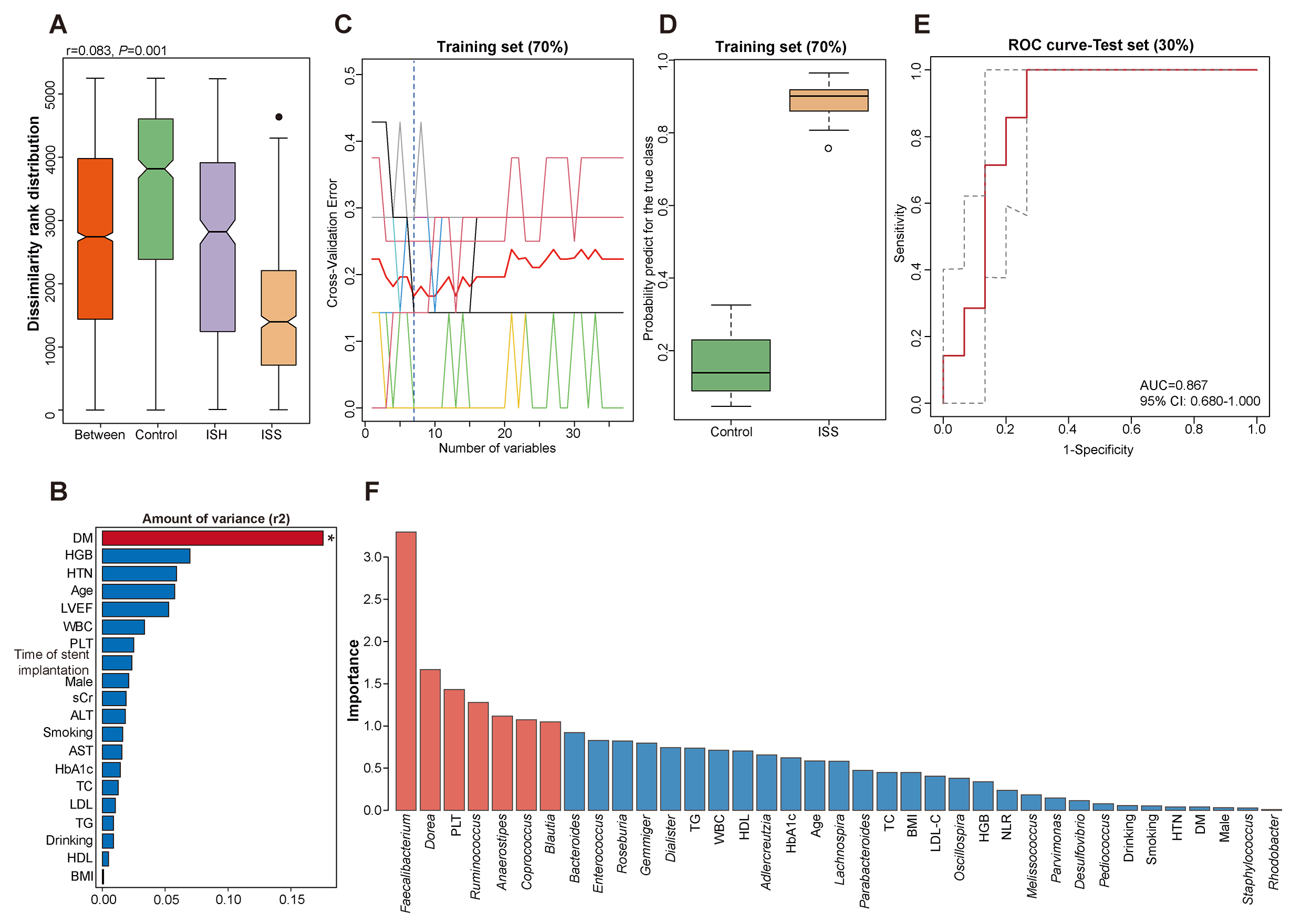

Supplement: Supplementary Figure S1 — Detailed information of ANOSIM, envfit and random forest analysis. [file Image_1.TIF]

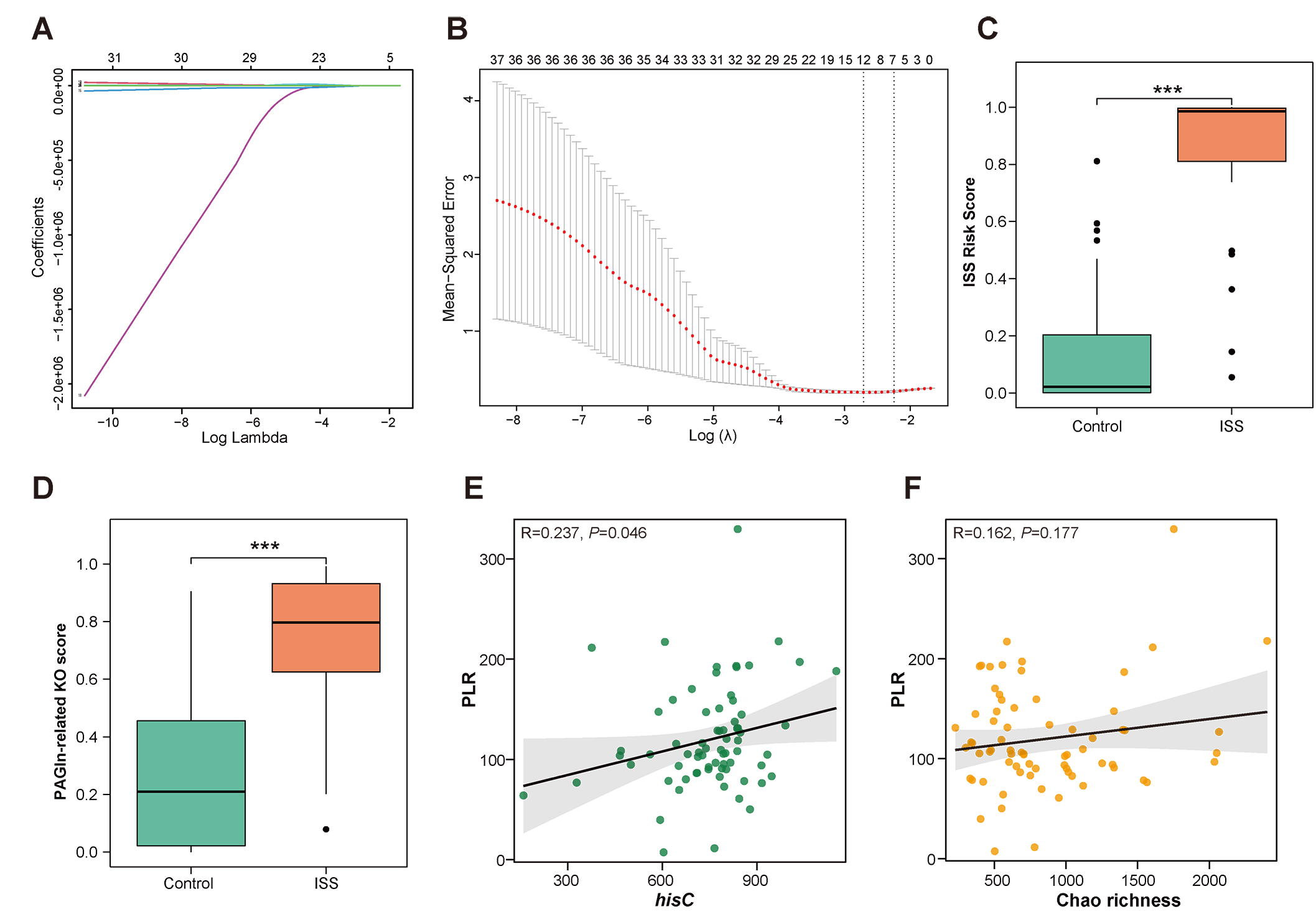

Supplement: Supplementary Figure S2 — Detailed information of LASSO analysis and correlation between PLR and hisC as well as Chao richness. [file Image_2.tif]
